# T313M polymorphism of the PINK1 gene in Parkinson’s disease

**DOI:** 10.3892/etm.2014.1702

**Published:** 2014-05-08

**Authors:** QIN LUO, XINLING YANG, YANI YAO, HONGJUAN LI, YULING WANG

**Affiliations:** 1 Department of VIP Integrated Medicine, The First Affiliated Hospital of Xinjiang Medical University, Urumqi, Xinjiang 830054, P.R. China; 2 Department of Rehabilitation, The People’s Hospital of Wenling, Wenling, Zhejiang 317500, P.R. China

**Keywords:** Uygur nationality, Han nationality, Parkinson’s disease, PINK1 gene, polymorphism

## Abstract

The present study aimed to investigate the association between T313M polymorphism at exon 4 of the PTEN-induced putative kinase 1 (PINK1) gene and Parkinson’s disease (PD) in the Uygur and Han populations of Xinjiang, China. Genetic DNA was extracted from 364 patients with PD from the Uygur and Han populations, as well as 346 normal control patients. Four exons of the PINK1 gene were amplified using quantitative polymerase chain reaction. The exons were then digested for restriction fragment length polymorphism analysis. Gene types and allele frequencies were identified using agarose gel electrophoresis followed by DNA sequencing to analyze the T313M polymorphisms. In the Han population, T313M polymorphism allele frequency was observed to be significantly different between the PD group and the control group (χ^2^=6.247; P<0.05). Significant differences were observed in in the T313M allele and genotype frequencies between the Uygur and Han populations (χ^2^=5.475 and χ^2^=10.950, respectively; *P*<0.05). Polymorphisms in the PINK1 T313M mutation may be associated with genetic susceptibility to PD.

## Introduction

Parkinson’s disease (PD) is a common neurodegenerative disease that is prevalent in 1–2% of individuals aged ≥65 years. While the specific etiology of PD is yet to be elucidated, genes involved in the development of the disease have attracted much research attention. At present, three autosomal recessive genes have been identified to be associated with PD, including PTEN-induced putative kinase 1 (PINK1), parkin, and DJ-1 (1–3). The PINK1 gene encodes a cytosolic E3 ubiquitin ligase and a mitochondrial serine/threonine kinase. PINK1 mutations were initially observed in consanguineous families of Italian and Spanish origin and are associated with slowly progressive PD with an onset prior to 50 years of age (4,5). PINK1 mutations have been reported in Filipino, Taiwanese, Israeli, Japanese, Irish and North American populations, and also in Chinese populations (6–11).

In order to investigate PINK1 mutations in PD and analyze the distribution of PINK1 gene T313M polymorphisms in the Uygur and Han populations of China, patients with PD and healthy individuals were investigated. The significance of PINK1 T313M polymorphisms in the pathogenesis of PD was also assessed.

## Materials and methods

### Diagnostic criteria

The present study was performed in accordance with British Brain Bank diagnostic criteria (12), with partial diagnoses and complex cases confirmed by a senior doctor from the Neurological Department of the First Affiliated Hospital of Xinjiang Medical University (Urumqi, China). Over the past 50 years, diagnostic criteria for early onset and late onset PD have been defined by developments in head magnetic resonance imaging and computed tomography examinations. The present study excluded patients with secondary PD, Parkinson-plus syndromes, nervous system disease, hyperthyroidism and other genetic diseases. The present study was conducted in accordance with the Declaration of Helsinki and was conducted with approval from the Ethics Committee of the first Affiliated Hospital of Xinjiang Medical University. Written informed consent was obtained from all participants.

### Patient data

In the present study, 364 patients with PD from the Uygur and Han populations of Xinjiang, China were selected between July 2010 and March 2011 as the case group. These patients included 175 individuals from the Uygur population, of which 99 were male and 76 were female, aged between 31 and 95 years (mean, 62.63±12.71 years). The remaining 189 patients with PD were from the Han population, of which 107 were male and 82 were female, aged between 25 and 85 years (mean, 61.76±12.31 years). The control group comprised 346 healthy individuals without PD. These included 163 individuals from the Uygur population, of which 92 were male and 71 were female, aged between 33 and 90 years (mean, 62.78±12.50 years), and 183 individuals from the Han population, of which 101 were male and 82 were female, aged between 27 and 86 years (mean, 61.43±12.51 years). No significant differences in age or gender were observed between the individuals in the PD group compared with those in the control group (χ^2^_gender_=0.048, P>0.05; t_age_=1.445, P>0.05).

### DNA extraction

Subsequent to obtaining informed consent, 2 ml venous blood was extracted from each patient. EDTA was used as an anti-coagulant and a blood extraction kit (Tiangen Biotech Co., Ltd., Beijing, China) was used to extract the DNA.

### Primers

Primer3 software was used to design the primer sequences required for polymerase chain reaction (PCR) analysis. The primer sequences for exon 4 of the PINK1 gene were as follows: 5′-GAATGTCAGTGC CAGTGTTGG-3′ (forward) and 5′-AGATATGTTCCCTTT GCATGGC-3′ (reverse). The length of amplified fragment was 429 bp and the primers were synthesized by Huada Gene Company (Beijing, China).

### Restriction fragment length polymorphism analysis

Restriction fragment length polymorphism analysis was performed using the *Hga*I endonuclease (New England Biolabs, Inc., Ipswich, MA, USA) in a restriction enzyme reaction system with a total reaction volume of 20 μl. The reaction consisted of 10 μl PCR product, 2 μl NEBuffer 1.1 (pH=8.0), 7.7 μl deionized double-distilled water and 0.3 μl *Hga*I, at 37°C overnight.

### Digested products confirmation

**Figure 1:**
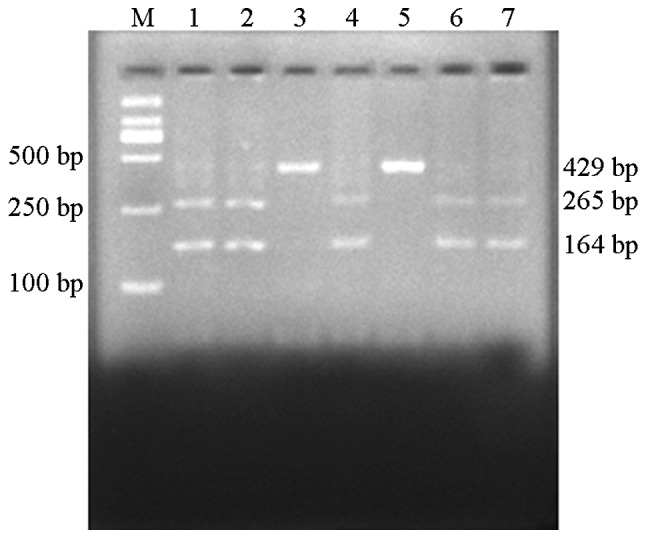
Genotypes of the PINK1 gene T313M polymorphism. Lanes 3 and 5 show homozygous mutant sequences which cannot be digested and remain as 429 bp fragments; type T/T. Lanes 1,2,4,6 and 7 show wild-type homozygous sequences that were digested into three fragments of 429, 265 and 164 bp; type C/T. M, 2,000 bp marker; PINK1, PTEN-induced putative kinase 1.

**Figure 2:**
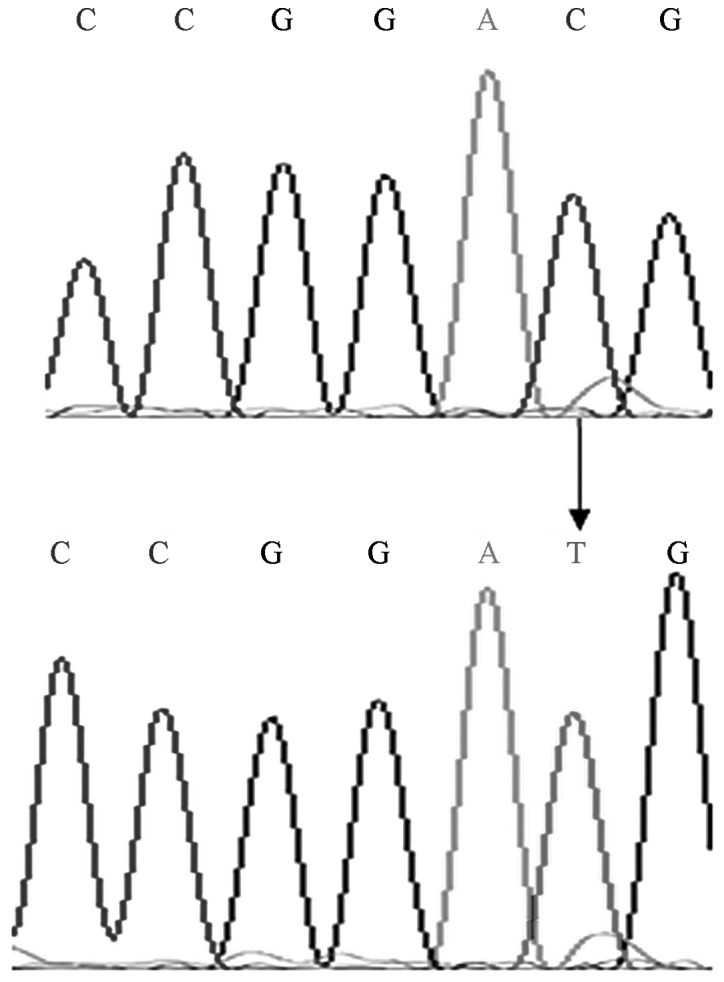
Sequencing of the fourth exon of the PINK1 gene in Parkinson’s disease shows a mutation from C to T at the 938 site. The sequence around the mutation site is shown, with the arrow pointing to the substitution. PINK1, PTEN-induced putative kinase 1.

Digested products were subjected to agarose gel electrophoresis and the enzymes were analyzed using UV gel electrophoresis. In brief, the gene amplified by PCR was a 429-bp sequence of the fourth exon of the PINK1 gene fragment, which contained a *Hga*I enzyme restriction site. Upon *Hga*I restriction digestion, homozygous wild-type sequences were digested into two fragments of 265 bp and 164 bp. This genotype was type C/C. At the 938 site, a C to T mutation occurs, resulting in loss of the *Hga*I restriction site. Thus, mutant homozygote genotypes are not digested and remain as 429 bp fragments, type T/T. Upon *Hga*I restriction digestion, heterozygous sequences are digested into fragments of 429, 265 and 164 bp. This heterozygous genotype is type C/T (Fig. 1). The mutant genotype was sequenced which confirmed that the digestion results were accurate (Fig. 2).

### Statistical analysis

Genotypes and allele frequencies were calculated using a direct counting method. Genotype data were subjected to Hardy-Weinberg equilibrium tests and alleles and genotypes were compared using the χ^2^ test. The constituent ratio of each group was analyzed using the χ^2^ test. All statistical analyses were performed using SPSS software, version 17.0 (SPSS, Inc., Chicago, IL, USA).

## Results

### Hardy-Weinberg equilibrium test

To show that the PINK1 genotype frequencies were equal in the case and control groups, the genotype frequencies of the case and control groups were assessed using alignment inspection. The PINK1 genotype distributions in the case and control groups were in Hardy-Weinberg equilibrium and exhibited good consistency (P>0.05).

### T313M allele frequency distribution

Differences in polymorphic T313M alleles and genotype frequencies in the PD group compared with the control group were not observed to be statistically significant (P>0.05; Table I). Furthermore, in the Uygur population, the genotype and allele frequencies in the PD group were not observed to be significantly different from those in the control group (P>0.05). Moreover, in the Han population, the genotype frequencies of the PD group showed no significant difference from those in the control group (P>0.05), whereas allele frequencies were observed to be significantly different between the PD and control groups (χ^2^=6.247, P<0.05; Table II).

When the Uygur and Han patients with PD were compared, the genotype and allele frequencies were observed to differ significantly between the two groups (χ^2^=5.475, χ^2^=10.950, P<0.05; Table III). When the study subjects were grouped according to age, (≤50 years and >50 years), the T313M polymorphisms in the PD group compared with the control group showed no significant difference (P>0.05; Table IV). Furthermore, when the study subjects were grouped according to gender, T313M polymorphisms in the PD group compared with the control group also showed no significant difference (P>0.05, Table V).

## Discussion

Mutations in the PINK1 gene on chromosome 1p36 have been reported in ~5% of patients with autosomal recessive PD (13). PINK1 mutations have been reported in patients with autosomal recessive early-onset PD (AREP) in Italy (14,15). The frequency of PINK1 mutations in patients with AREP is between 2.9 and 29%, with the mutation frequency varying greatly depending on ethnicity (16–20). In patients with sporadic early-onset PD (EOP), the pathogenic role of PINK1 mutation is particularly important (13,21). The mutation frequency range is wide. PINK1 gene mutation has not been observed in patients with sporadic PD in the USA (22). Furthermore, among a Chinese population in Taiwan, only one heterozygous PINK1 gene mutation was found in 73 patients with sporadic EOP (22). In Italy, patients with sporadic EOP have been reported to have relatively high mutation rates (14). Klein *et al* (15) observed that PINK1 gene mutations in patients with sporadic EOP were similar to those in the parkin gene (15). At present, few reports on minority PD genes are available, particularly concerning the PINK1 T313M mutation in populations in Xinjiang, China.

The present study showed that genotype frequencies and allele frequency distributions were not significantly different between patients with sporadic PD and healthy control individuals in the total study population. However, in patients with PD, the distribution of T313M polymorphisms was significantly different between patients from the Uygur and Han populations, and all were homozygous mutants. These findings suggest that differences in gene polymorphisms may exist between patients with PD from Uygur and Han populations. These findings are inconsistent with those of Guo *et al* (23) and Zhang et al (24), who identified that in 120 patients with sporadic PD, as well as in patients with pedigree mutations, mutations may not only exist in patients with familial PD, but also those with sporadic PD in the Han population. However, the sample size should be further expanded and uneven sampling may be associated with the results observed. In the present study, in the Uygur populations, the allele frequencies of the T313M polymorphism in the PD group were not found to be significantly different from those in the control group. However, in the Han population, the allele frequencies were observed to be significantly different in the PD group compared with the control group. These findings differ from those obtained in other regions of mainland China (25). When grouped according to age and gender, the PD and control groups showed no statistically significant difference. Further expansion of the sample size of the patients with early-onset PD is necessary to verify these findings. It has previously been reported that homozygous or compound heterozygous PINK1 mutations are more common in patients with AREP and single heterozygous mutations are found in patients with sporadic EOP (19,26,27). The present study showed that in the Xinjiang area, T313M polymorphism of the PINK1 gene in the PD and control groups was a homozygous mutation. Significant differences were found between the Uygur and Han populations; the T allele frequency was 2.9% in the Ugyur patients with PD, whereas the T allele was absent from the Han patients with PD, suggesting that T313M gene polymorphism was related to nationality. The Uygur population may be expected to have an increased prevalence of PD due to the incidence of PINK1 mutations. However, no significant difference was observed between the PD group and the control group, and the frequency of the type T allele (1.4%) was not found to increase PD prevalence. In the present study, no significant difference was observed in the allele and genotype frequencies of the PINK1 gene T313M polymorphism in patients aged ≤50 years compared with those aged >50 years. This finding suggests that T313M may not be a predisposing factor of early-onset PD.

The present study identified that differences in T313M polymorphisms exist between the Uygur and Han populations. The PINK1 gene may be associated with genetic susceptibility to PD, particularly in the Han population, which may be associated with the particular area of Xinjiang, living environment and genetic background. Furthermore, sampling may be not uniform. Further investigations, including a larger sample size, are required to validate these results.

## Figures and Tables

**Table I:** Allele and genotype frequencies of the PINK1 gene T313M polymorphism in patients with PD and healthy control individuals.

	Genotype frequency			Allele frequency		
		
Groups	No. of cases	T/T, n (%)	C/T, n (%)	C/C, n (%)	T type, n (%)	C type, n (%)
PD	364	5 (1.4)	0 (0.0)	359 (98.6)	10 (1.4)	718 (98.6)
Control	346	5 (1.4)	0 (0.0)	341 (98.6)	10 (1.4)	682 (98.6)

PD, Parkinson’s disease; PINK1, PTEN-induced putative kinase 1.

**Table II:** Allele and genotype frequencies of the PINK1 gene T313M polymorphism in patients with PD from Uygur and Han populations compared with healthy control individuals.

		Genotype frequency			Allele frequency	
			
Groups	No. of cases	T/T, n (%)	C/T, n (%)	C/C, n (%)	T type, n (%)	C type, n (%)
Uygur population						
PD	175	5 (2.9)	0 (0.0)	170 (97.1)	10 (2.9)	340 (97.1)
Control	163	2 (1.2)	0 (0.0)	161 (98.8)	4 (1.2)	322 (98.8)
Han population						
PD	189	0 (0.0)	0 (0.0)	189 (100.0)	0 (0.0)	378 (100.0)
Control	183	3 (1.6)	0 (0.0)	180 (98.4)	6 (1.6)	360 (98.4)

PD, Parkinson’s disease; PINK1, PTEN-induced putative kinase 1.

**Table III:** Allele and genotype frequencies of the PINK1 gene T313M polymorphism in patients with PD from Uygur and Han populations.

		Genotype frequency			Allele frequency	
			
Groups	No. of cases	T/T, n (%)	C/T, n (%)	C/C, n (%)	T type, n (%)	C type, n (%)
Uygur with PD	175	5 (2.9)	0 (0.0)	170 (97.1)	10 (2.9)	340 (97.1)
Han with PD	189	0 (0.0)	0 (0.0)	189 (100.0)	0 (0.0)	378 (100.0)

PD, Parkinson’s disease; PINK1, PTEN-induced putative kinase 1.

**Table IV:** Association between age and allele and genotype frequencies of the PINK1 gene T313M polymorphism in patients with PD and healthy individuals

			Genotype frequency			Allele frequency	
				
Age	Group	No. of cases	T/T, n (%)	C/T, n (%)	C/C, n (%)	T type, n (%)	C type, n (%)
≤50 years	PD	80	1 (1.3)	0 (0.0)	79 (98.8)	2 (1.3)	158 (98.8)
	Control	74	0 (0.0)	0 (0.0)	74 (100.0)	0 (0.0)	148 (100.0)
>50 years	PD	284	4 (1.4)	0 (0.0)	280 (98.6)	8 (1.4)	560 (98.6)
	Control	285	5 (1.8)	0 (0.0)	280 (98.2)	10 (1.8)	560 (98.2)

PD, Parkinson’s disease; PINK1, PTEN-induced putative kinase 1.

**Table V:** Association between gender and allele and genotype frequencies of the PINK1 gene T313M polymorphism in patients with PD and healthy individuals.

			Genotype frequency			Allele frequency	
				
Gender	Group	No. of cases	T/T, n (%)	C/T, n (%)	C/C, n (%)	T type, n (%)	C type, n (%)
Male	PD	206	3 (1.5)	0 (0.0)	203 (98.5)	6 (1.5)	406 (98.5)
	Control	193	1 (0.5)	0 (0.0)	192 (99.5)	2 (0.5)	384 (99.5)
Female	PD	158	2 (1.3)	0 (0.0)	156 (98.7)	4 (1.3)	312 (98.7)
	Control	153	4 (2.6)	0 (0.0)	149 (97.4)	8 (2.6)	298 (97.4)

PD, Parkinson’s disease; PINK1, PTEN-induced putative kinase 1.
